# Habitat and social context affect memory phenotype, exploration and covariance among these traits

**DOI:** 10.1098/rstb.2017.0291

**Published:** 2018-08-13

**Authors:** Sarah Dalesman

**Affiliations:** Institute of Biological, Environmental and Rural Sciences, Aberystwyth University, Penglais, Aberystwyth, Ceredigion SY23 3DA, UK

**Keywords:** animal personality, behavioural syndrome, cognition, exploration, memory, stress

## Abstract

Individual differences in cognitive ability are predicted to covary with other behavioural traits such as exploration and boldness. Selection within different habitats may act to either enhance or break down covariance among traits; alternatively, changing the environmental context in which traits are assessed may result in plasticity that alters trait covariance. Pond snails, *Lymnaea stagnalis*, from two laboratory strains (more than 20 generations in captivity) and F1 laboratory reared from six wild populations were tested for long-term memory and exploration traits (speed and thigmotaxis) following maintenance in grouped and isolated conditions to determine if isolation: (i) alters memory and exploration; and (ii) alters covariance between memory and exploration. Populations that demonstrated strong memory formation (longer duration) under grouped conditions demonstrated weaker memory formation and reduced both speed and thigmotaxis following isolation. In wild populations, snails showed no relationship between memory and exploration in grouped conditions; however, following isolation, exploration behaviour was negatively correlated with memory, i.e. slow-explorers showing low levels of thigmotaxis formed stronger memories. Laboratory strains demonstrated no covariance among exploration traits and memory independent of context. Together these data demonstrate that the relationship between cognition and exploration traits can depend on both habitat and context-specific trait plasticity.

This article is part of the theme issue ‘Causes and consequences of individual differences in cognitive abilities’.

## 1. Introduction

Consistent individual differences in behavioural traits, either across time or contexts in the same trait (animal personality) or across suites of traits (behavioural syndrome), have now been demonstrated in a diverse range of taxa (e.g. see reviews in [1,2]). Individuals tend to differ on a behavioural continuum, often described as ranging from bold to shy or proactive to reactive, thought to be linked to differences in underlying physiology [3]. Covariance among and consistency within behavioural traits are considered to play an important role in the ecology and evolution of behaviour [4,5]. An emerging area from this work is determining the role that consistent individual differences may play in cognition, considered here as the perception, acquisition, processing, storage and use of information [6]. For example, personality could influence learning style (speed and accuracy of information acquisition) [7], or cognition may influence behavioural consistency affecting how animals respond to their environment [8,9].

Individual differences in behaviour are predicted to covary with the way in which animals perform in cognitive tasks. For example, exploratory behaviour is one aspect often used in determining the link between behaviour and cognitive ability. Fast explorers are predicted to acquire information about their environment more rapidly [9], and there is an indication that fast-exploring individuals also show a greater tendency to engage with testing apparatus [10,11]. However, fast-exploring individuals may also be less accurate in the information they acquire [7] and demonstrate less flexibility in altering behaviour in response to change once information is acquired [9]. Studies so far indicate considerable variation both between and within species in the relationship between exploration and cognitive performance, with no clear pattern emerging (reviewed in [12]). For example, among-species, fast-exploring goats demonstrated slower acquisition of a visual discrimination task [13], whereas fast-exploring cavies were quicker to learn object discrimination [14]. Within-species, exploratory behaviour does not appear related to speed of acquiring information in instrumental discrimination, colour association or detour-reaching tasks in black-capped chickadees; however, slow-explorers showed greater accuracy during recall [15]. This contrasts with earlier work showing that slow-exploring black-capped chickadees are also slower to learn an acoustic discrimination task [16]. Therefore, ability to perform a cognitive task may not be due to underlying ability to learn and form memory *per se*, but instead a consequence of non-cognitive differences among individuals [8]. How these non-cognitive differences affect speed of acquisition and efficacy of recall will be highly dependent on the nature of the cognitive task assessed.

Context may impact on covariance between cognitive and behavioural traits. Environmental conditions can enhance or breakdown non-cognitive behavioural consistency (reviews in [17,18]), which may occur through cognitive processes, for example as the animal learns about aspects of its environment. Populations from different habitat types often express differences in cognitive ability across a range of species, including Hymenoptera [19,20], fish [21] and birds [22], suggesting selection on cognitive traits within specific environmental conditions. Environment can also impact on cognition through intrinsic changes such as plasticity in brain structure [23] or alterations to the gut microbiome [24]. The context in which cognition is assessed can impact on traits: for example, effects of stress on learning and memory are found across species (e.g. [25,26]) and stress is considered a key modulator of behavioural integration with other phenotypic traits [27]. Within the same species, populations or strains may differ in their cognitive ability [28,29] and in how they respond to stress [30–32]. While the effects of population differences and experimental context have been tested across multiple species in both cognitive and non-cognitive traits, the effect on the behaviour–cognition relationship has not been determined.

The pond snail, *Lymnaea stagnalis*, provides an ideal model system to investigate how population differences and environment impact on trait covariance between behaviour and cognition. Cognitive ability can be tested through memory formation under highly controlled conditions where all individuals receive relevant stimuli. This eliminates a major issue that may occur in relating cognitive ability to personality, where experience of relevant stimuli is dependent on voluntary participation in a task and the level of participation relates to animal personality. Memory across a range of traits has been shown to both differ significantly among populations and also show covariance at an individual level [28]. Additionally, memory is affected by a range of environmentally relevant stressors [33], so the context in which behaviour and cognition are tested can be easily altered. Social isolation over relatively acute periods (one week) has been demonstrated to alter mating behaviour [34] and also exploration behaviour [35]. Therefore, social condition was chosen as a context in which to test the relationship between exploration and cognition, using operant conditioning of aerial respiration as a proxy for memory phenotype [28] and exploration behaviour, measured as crawling speed (giving an indication of overall activity) and degree of thigmotaxis in a novel behavioural arena [35,36]. Snails from two laboratory strains and six wild populations were assessed for memory formation under either grouped or isolated conditions. The within-individual effect of isolation on exploration behaviour was assessed and compared with memory phenotype under grouped conditions to determine whether the relationship between exploration and memory differed depending on social context during exploration. It was predicted *a priori* that populations or strains would differ significantly in their ability to demonstrate memory formation in grouped conditions [28], and that those populations demonstrating stronger memory formation would be less affected by isolation due to a resistance to the effects of environmental stress [30]. It was also predicted that memory would covary with locomotion under grouped conditions as both are likely to be directly related to metabolic rate [37] as seen in response to low calcium stress [38,39], but the effect of social isolation on exploration would break down this covariance.

## 2. Methods

Adult (25 ± 1 mm spire height) pond snails, *L. stagnalis*, were sourced from eight separate populations previously shown to differ in memory phenotype [28]. Two were ‘laboratory’ strains having been maintained in laboratory conditions for at least 20 generations (L1 and L2), four populations were sourced from rivers (R1–R4) and two from ditch sites (D1 and D2) on the Somerset Levels, UK, using F1 generation animals to carry out experiments [28]. All animals were reared under standard laboratory conditions in oxygenated artificial pond water containing 80 mg l^−1^ Ca^2+^ [28]. Snails were kept at room temperature (20 ± 1°C) at a stocking density of two snails per litre in 6 l aquaria (standard grouped conditions) on a 14:10 light:dark schedule and fed lettuce ad libitum. Trout pellets were also added once per week to provide an additional source of protein. Different cohorts of snails were used in each of experiments 1–3. All snails were labelled using queen bee tags (E. H. Thorne Ltd, UK) attached to the shell with non-toxic Loctite 454 adhesive (Henkel, UK) to track individual behaviour throughout the experiments.

### (a) Experiment 1: Confirmation of memory variation among populations

Long-term memory (LTM) following operant conditioning of aerial respiration was assessed in populations previously identified to vary in memory formation [28]. Snails were maintained in standard grouped conditions throughout experiment 1. During contingent operant conditioning, the snail was gently poked on a breathing orifice (the pneumostome) when it tried to perform aerial respiration (see electronic supplementary material for detailed methods of operant conditioning). Non-contingent yoked controls were used to confirm that changes in behaviour were due to operant association rather than general sensitization. During the test 24 h following the first training trial, snails were poked contingently on the pneumostome for all groups. Memory was considered to have formed if the snails showed a significant decrease in breathing attempts during the test compared to the first training session. Two training protocols were used, a single half-hour training session (*N* = 206, 10–16 per group × population) and two half-hour training sessions (*N* = 188, 11–13 per group × population), with a memory test for LTM at 24 h following training. The former was predicted to result in LTM formation in half the populations tested [28], with only four of the populations predicted to demonstrate strong memory formation (LTM following a single training session), whereas the latter results in LTM formation in all populations [33].

### (b) Experiment 2: Impact of isolation on memory formation

To determine the effect of isolation on memory, three groups were compared: (i) maintained in standard grouped conditions and trained in grouped conditions; (ii) maintained in standard grouped conditions and trained in isolation; and (iii) maintained in isolation and trained in isolation (see electronic supplementary material, figure S1; *N* = 330, 11–16 per treatment group × population). Isolated snails were held in individual 500 ml perforated containers for one week prior to testing, with 12 containers per aquarium in 6 l of aerated pond water. This period of isolation has been shown to be sufficient to cause changes in exploration [35]. All snails were trained contingently as outlined in experiment 1 using two training sessions, which typically results in LTM in all populations. The second training session was also used to test for intermediate-term memory (ITM) following isolation to confirm that all populations had learnt (see electronic supplementary material). Snails were tested for LTM 24 h following the first training trial.

### (c) Experiment 3: Impact of isolation on covariance between exploration and memory

LTM following a single training session in grouped conditions (as in experiment 1) was compared with exploration behaviour following maintenance in both grouped and isolated conditions in the same individuals. A single training session was used as this had previously been determined to result in among-population variability in memory formation at 24 h [28]. The goal was to determine whether environmental effects on variation in exploration, a commonly used measure of animal personality, altered how this trait relates to cognitive ability. Individual snails from each population (*N* = 152, 18–20 per population) were tested for each element (memory and exploration) in a randomized block design, whereby they were randomly assigned to receiving either memory or exploration trials first, and either grouped or isolated prior to testing exploration (four possible combinations; electronic supplementary material, figure S2). All individuals were given a final locomotion trial one week following return to standard grouped conditions at the end of the experiment to determine overall consistency in exploration behaviour.

To assess exploration, snails were placed individually into a 150 mm Falcon^®^ culture dish with a 2 × 2 cm grid marked on the base containing 200 ml standard pond water. Once the snail had emerged (tentacles and eyes fully visible) their movement within the arena was tracked for 15 min. This allowed calculation of the speed of locomotion (distance travelled over 15 min to give speed in mm s^−1^) as well as the proportion of time during the 15 min period that snails spent in contact with the arena edge to determine thigmotaxis. Since snails primarily rely on chemoreception in investigating their environment [40], thoroughly cleaning the arena between individuals with alcohol resulted in the arena appearing to be a novel environment to the snail on encountering it each time.

#### (i) Data analyses

All data were analysed using analysis of variance (ANOVA) or Pearson's correlations in SPSS 24.0 (SPSS Inc., Chicago, IL, USA). Sidak *post hoc* comparisons (*α* = 0.05) were used to determine pair-wise differences. The Satterthwaite approximation was used to estimate degrees of freedom. Effect size is presented as 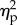. Homogeneity of variance was confirmed for ANOVA. Correlation data were checked for linearity, homoscedasticity and presence of outliers prior to analysis.

To determine LTM formation, the relative change in breathing rate between the first training session (TR1) and testing (MT) was used in analyses (TR1 − MT). The change in behaviour between the first and second training sessions (TR1 − TR2) was used to determine ITM formation. Data were then converted to proportional change in breathing attempts relative to the initial breathing rate during TR1 to account for initial individual differences in breathing rate.

Experiment 1: LTM formation was assessed following both single and two training trials, using training regime (contingent versus yoked) and habitat of origin (laboratory versus ditch versus river) as fixed factors and population as a random factor nested in habitat in the model.

Experiment 2: To determine effects of isolation on memory, initial breathing rate, ITM and LTM were compared using habitat of origin (laboratory versus ditch versus river) and maintenance condition (grouped, isolated during training only and isolated for one week) as fixed factors, and population as a random factor nested in habitat in the model.

Experiment 3: Pearson's correlations were used to determine covariance in exploration over the two grouped trials, i.e. determining if exploration is consistent over time in the absence of isolation. Memory was converted to a positive value (higher number = greater reduction in breathing attempts) and the proportional change in breathing attempts relative to the initial breathing rate during TR1 was used for analyses to account for initial individual differences in breathing rate. The relationship between memory formation and exploration traits, crawling speed and thigmotaxis was assessed separately for laboratory, ditch and river populations following maintenance in both grouped and isolated conditions. Habitat was predicted to influence covariance among traits based on previous work [28].

The response of exploration traits to isolation was analysed to determine whether changes in speed and thigmotaxis were dependent on habitat and memory phenotype (see electronic supplementary material, Plasticity in exploration traits, for details).

## 3. Results

### (a) Experiment 1: Confirmation of memory variation among populations

**Figure 1.**
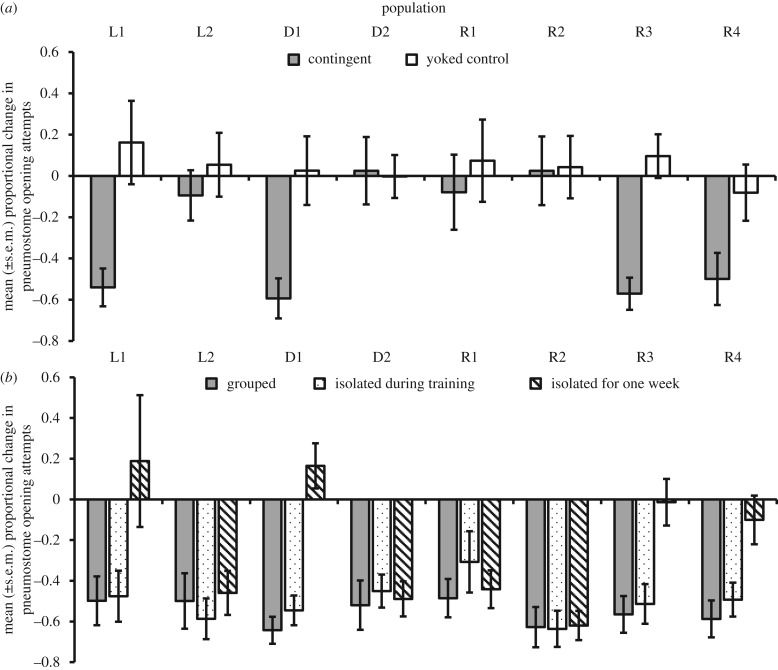
Mean proportional change in pneumostome opening attempts between training and test conditions across eight populations: (*a*) following contingent or yoked single-trial training; and (*b*) following two-trial training either grouped throughout, isolated during training only or isolated for a week.

Following two training sessions, only contingently trained animals demonstrated a reduction in breathing behaviour (ANOVA: main effect of training: *F*_1,5.23_ = 582.006, *p* < 0.001, 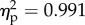; difference between contingent and yoked training = −0.724; CI: −0.869, −0.578; electronic supplementary material, table S1). There was no difference in response among populations (electronic supplementary material, table S1): all populations demonstrated memory following contingent training, but not following yoked training. However, following a single training session, the response to training differed among populations (ANOVA: training × population(habitat): *F*_5,190_ = 2.789, *p* = 0.019, 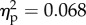), with only half the populations showing a significant difference between contingent and yoked training (figure 1*a*: Sidak pair-wise comparisons within population: *p* < 0.05 for D1, L1, R3 and R4; *p* > 0.05 for D2, L2, R1 and R2: electronic supplementary material, tables S1 and S2). Habitat of origin did not affect the response to training in either the single or two training sessions' experiments (electronic supplementary material, table S1).

### (b) Experiment 2: Impact of isolation on memory formation

All populations demonstrated ITM, with no significant differences among populations or treatment (electronic supplementary material, table S3 and figure S3*a*), demonstrating that all populations learnt to alter breathing attempts during the initial training trial. Ditch and river snails showed a trend towards a greater proportional decrease in breathing attempts compared to laboratory snails (ANOVA: habitat: *F*_2,4.68_ = 9.644, *p* = 0.022, 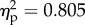), but pair-wise differences were not significant among treatment groups (Sidak: *p* > 0.50 for all pair-wise comparisons; electronic supplementary material text: Intermediate-term memory).

Isolation had a significant effect on LTM formation, but this was dependent on the population of origin (ANOVA: isolation × population(habitat): *F*_10,306_ = 3.256, *p* = 0.001, 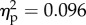; electronic supplementary material, table S3). Snails from half the populations failed to demonstrate LTM after one-week isolation (figure 1*b*; electronic supplementary material, table S4), whereas all populations demonstrated LTM in grouped conditions and following isolation during training only (figure 1*b*). The snails' habitat of origin did not significantly affect memory formation (electronic supplementary material, table S3).

There was no effect of isolation, habitat or population on initial breathing rate during the first training trial (electronic supplementary material, table S3); therefore, differences in memory were not due to differences in the number of stimuli (pokes) an individual received during operant conditioning.

### (c) Experiment 3: Impact of isolation on covariance between exploration and long-term memory

Snails were highly consistent in exploration traits between the two trials carried out following grouped maintenance (crawling speed grouped trial 1 versus grouped trial 2: *r*_P_ = 0.766, CI 0.677, 0.844, *p* < 0.001; thigmotaxis grouped trial 1 versus grouped trial 2: *r*_P_ = 0.627, CI 0.506, 0.732, *p* < 0.001). There was no consistency in exploration traits between the two social contexts for thigmotaxis (thigmotaxis grouped trial 1 versus isolated: *r*_P_ = 0.086, CI −0.084, 0.248, *p* = 0.293), whereas faster snails in grouped conditions remained faster following isolation (speed grouped trial 1 versus isolated: *r*_P_ = 0.252, CI 0.084, 0.45, *p* = 0.002).

**Figure 2.**
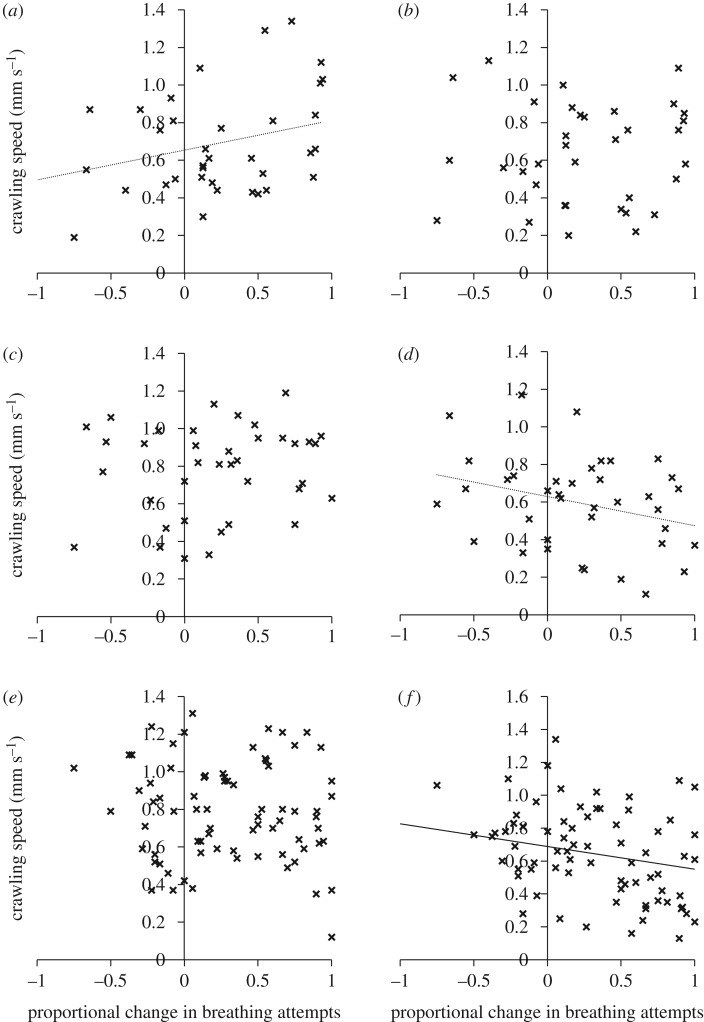
Relationship between crawling speed (mm s^−1^) and memory formation as proportional change in breathing attempts between the first training session and long-term memory test (converted to a positive value: higher value = greater decline in breathing attempts) in: (*a*) grouped laboratory strains; (*b*) isolated laboratory strains; (*c*) grouped ditch populations; (*d*) isolated ditch populations; (*e*) grouped river populations; and (*f*) isolated river populations. Trend lines are placed on the figure where the relationship is significant (solid line: *p* < 0.05) or show a non-significant trend (dotted line: *p* < 0.10).

**Figure 3.**
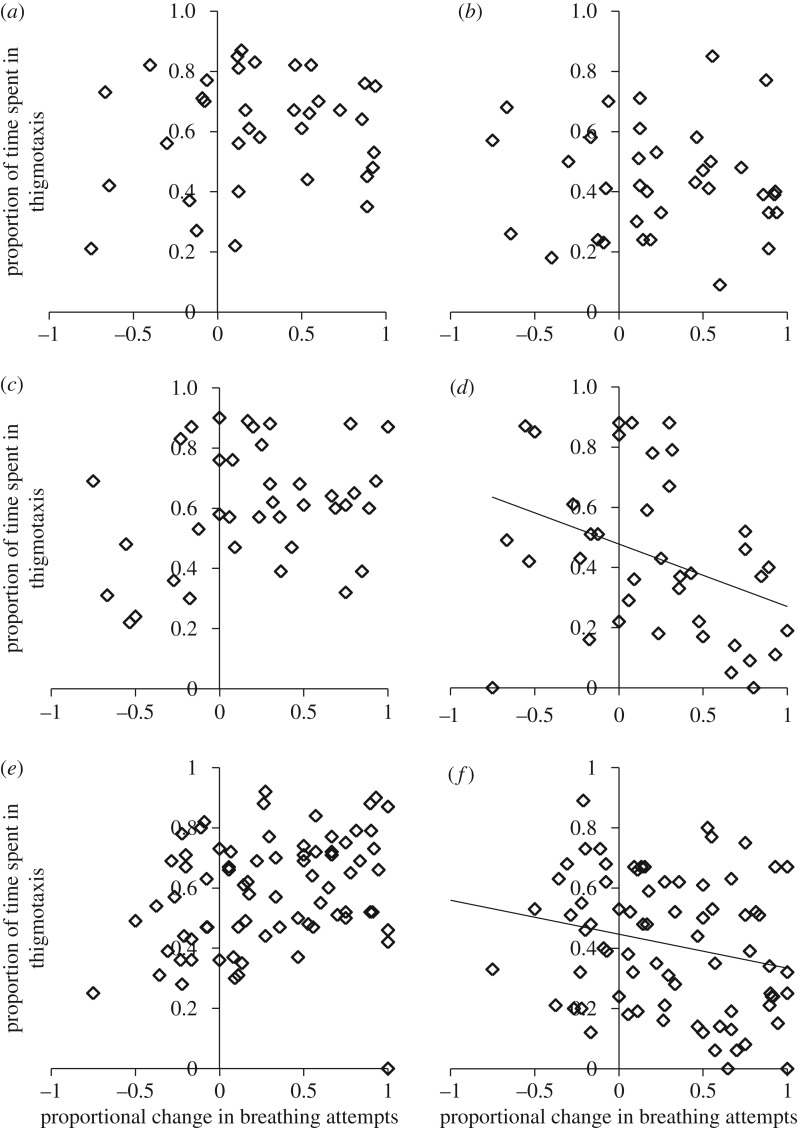
Relationship between thigmotaxis (proportion of time in contact with the arena wall) and memory formation as proportional change in breathing attempts between the first training session and long-term memory test (converted to a positive value: higher value = greater decline in breathing attempts) in: (*a*) grouped laboratory strains; (*b*) isolated laboratory strains; (*c*) grouped ditch populations; (*d*) isolated ditch populations; (*e*) grouped river populations; and (*f*) isolated river populations. Trend lines are placed on the figure where the relationship is significant (solid line: *p* < 0.05).

The relationship between exploration traits and strength of memory formation (proportional reduction in breathing attempts) depended on the social context and the habitat type that snails originated from. In grouped conditions, speed showed a non-significant trend towards positive covariance with memory in laboratory strains (figure 2*a*; *r*_P_ = 0.311, CI 0.010, 0.568, *p* = 0.065), but there was no relationship between speed and memory in ditch or river populations (figure 2*c*,*e*; ditch: *r*_P_ = 0.147, CI −0.175, 0.443, *p* = 0.380; river: *r*_P_ = −0.091, CI −0.282, 0.095, *p* = 0.427). In grouped conditions, thigmotaxis was not correlated with memory in any of the populations (figure 3*a*,*c*,*e*; laboratory: *r*_P_ = 0.122, CI −0.231, 0.416, *p* = 0.478; ditch: *r*_P_ = 0.271, CI −0.103, 0.560, *p* = 0.099; river: *r*_P_ = 0.125, CI −0.079, 0.497, *p* = 0.277).

Following isolation, laboratory strains showed no relationship between exploration traits and memory formation (figures 2*b* and 3*b*; speed: *r*_P_ = 0.042, CI −0.367, 0.323, *p* = 0.808; thigmotaxis: *r*_P_ = 0.028, CI −0.350, 0.281, *p* = 0.873). Ditch populations showed a non-significant trend towards negative relationship between memory formation and speed (figure 2*d*; *r*_P_ = −0.297, CI −0.538, 0.011, *p* = 0.070) and a negative relationship between memory formation and thigmotaxis (figure 3*d*; *r*_P_ = −0.361, CI −0.640, −0.008, *p* = 0.026). River populations showed a negative relationship between both exploration traits and memory formation (figures 2*f* and 3*f*; speed: *r*_P_ = −0.348, CI −0.523, −0.191, *p* = 0.002; thigmotaxis: *r*_P_ = −0.341, CI −0.487, −0.139, *p* = 0.002).

The change seen in the relationship between exploration traits and memory was the result of plasticity in exploration traits following isolation, which differed depending on both the memory phenotype and habitat of origin (see electronic supplementary material: Plasticity in exploration traits, table S5 and figure S4).

## 4. Discussion

All snail populations tested demonstrated long-term memory (LTM) of operant conditioning following two training sessions, whereas only half the populations tested demonstrated LTM following a single training session. Whether or not populations demonstrated LTM following single-trial training did not differ among habitats, with those from laboratory strains, ditches or rivers being equally likely to demonstrate LTM as found in previous work [28]. Isolation during training alone did not alter LTM formation; however, following maintenance in isolation for one week, populations that had demonstrated LTM following single-trial training failed to demonstrate LTM following two training sessions. Conversely, those populations that had failed to demonstrate LTM after a single training session apparently remained unaffected by isolation following two-trial training and demonstrated LTM at 24 h. This effect was not due to an inability to learn, as all populations demonstrated intermediate-term memory (ITM) irrespective of social condition. Many species demonstrate negative effects of social isolation on cognitive function, from invertebrates to humans [41,42]. The data presented here indicate that the populations that form LTM following a single training session under grouped conditions are more sensitive to social isolation, potentially acting as a stressor blocking their ability to form LTM. Similar results have been found in *Drosophila*, where carriers of the *for^s^* gene are typically better at demonstrating long-term memories compared to carriers of the *for^R^* gene [43], and also demonstrate greater sensitivity to the social environment in cognitive tasks [31]. It was predicted *a priori* that snail populations that typically fail to form LTM following a single training trial would be more susceptible to the effects of stress, as seen in previous work on the effect of low calcium availability on memory formation [30,38]. However, it appears that not all stressors can be considered equal from a snail's perspective, and social stress is perceived and/or responded to in a different manner from resource restriction. Whether the effect of social isolation interferes with perception of the physical stimulus during training, LTM formation *per se*, or ability to recall that information is yet to be determined.

Snail populations that demonstrate greater sensitivity to social isolation did not differ in baseline activity levels under grouped conditions. Therefore, differences in response to the social environment cannot be explained by experience related to variation in social interactions driven by baseline activity levels, as proposed for *Drosophila* [31]. However, following isolation, those populations that demonstrate LTM following single-trial training under grouped conditions do exhibit a change in exploratory behaviour, reducing speed and time spent in thigmotaxis. Changes in activity levels following isolation have been found in other species, typically resulting in a reduction in average activity levels [44,45]. A reduction in thigmotaxis is often considered to indicate a reduction in stress [46] and correlates with increased performance in cognitive tasks in other species [47,48]. Animals displaying reduced thigmotaxis explore their environment more efficiently; therefore, this may indirectly impact cognitive performance in spatial tasks in particular [47]. In *L. stagnalis*, the period of isolation used here increases desire to mate, particularly in the male role [34], and previous work has shown this also increases trail-following behaviour [35]. Therefore, a reduction in thigmotaxis in *L. stagnalis* is unlikely to be a direct response to reduced stress as seen in rodents and fish for example, but instead a result of increased exploration of a novel environment to locate conspecifics. Despite changes in exploration traits following isolation, the baseline breathing rate (i.e. the number of stimuli received during training) did not differ among different treatment groups when assessing the impact of isolation on memory. This suggests that the response of populations that fail to demonstrate memory following social isolation may be due to changes in either the perception of stimuli or direct impacts of isolation on neural plasticity in breathing behaviour preventing gene transcription necessary for LTM formation.

Covariance at an individual level among behavioural and cognitive traits has been highlighted as an important area in current research towards developing an understanding of the evolution of cognitive traits [9,49,50]. Here, covariance between exploration behaviour and memory formation was altered by the effect of isolation on exploration. Under grouped conditions, there was little evidence of a relationship between exploration and memory formation; however, following isolation, exploration traits were negatively correlated with memory formation in snails originating from ditch and river populations. Snails that formed stronger memories crawled more slowly and showed reduced thigmotaxis. The impact of changing the environment on trait covariance is not unexpected, as the environment has been found to alter covariance among non-cognitive traits in other species [18,51,52]. However, isolation was predicted *a priori* to break down rather than enhance covariance among exploration and memory traits. Context may also impact on covariance among memory traits: for example, bumblebees, *Bombus terrestris*, demonstrate positive covariance in associative memory formation using odour or visual cues when freely foraging [53], but no relationship between associative memories of odour and visual cues when odour is tested under restraint [54]. This could be due to differences in the cognitive processing requirements between free-flying and restrained tasks, but could also be due to altering the context in which the animals were tested, i.e. that restraint may act as a stressor for the bees.

Habitat did not impact on whether snails demonstrated LTM in grouped or isolated conditions; however, habitat of origin did play an important role in determining both changes in exploration following isolation and strength of covariance between memory and exploration. Laboratory strains stand out as neither strain shows covariance between memory phenotype and speed or thigmotaxis following isolation. Similar results were found in testing covariance across different memory traits in *L. stagnalis* where no evidence of covariance was found in laboratory populations, but covariance among memory traits was found in F1 snails from wild populations [28]. All populations used were reared under common garden conditions at similar densities, so it is unlikely that differences in social experience played a role in individual differences in trait covariance. Transgenerational effects may alter trait covariance as it can alter a wide range of offspring behaviours, particularly through stress experienced by the parental generation [55]. The ditch and river populations were the offspring of wild-caught adults experiencing a wide range of potential environmental stressors compared to rearing in the laboratory with no predation threat, plentiful mating opportunities and food ad libitum. More than 20 generations in the laboratory may also have relaxed selection on covariance among traits [56]. Further work on selection of traits or exposure of the parental generation to unpredictable environments in the laboratory may elucidate which of these factors drive changes following multiple generations in captivity.

## 5. Conclusion

The data presented here clearly demonstrate that context may strongly influence our conclusions about relative cognitive abilities within species: populations that formed LTM following single-trial training under grouped conditions were the same populations that failed to form LTM following isolation. This is highlighted in a recent review by Rowe & Healy [49], which asserts that non-cognitive factors need to be considered when assessing variation among individuals both within and among species. These data also clearly demonstrate that, like behavioural syndromes [57], covariance between behavioural and cognitive traits may not be stable across environmental contexts or habitat type. Therefore, we need to consider both the origin of the animals used and the context in which they are tested to understand the role that the relationship between non-cognitive behavioural traits and cognition may play in selection on cognition.

## Supplementary Material

Supplementary methods and results

## Supplementary Material

Data

## Data accessibility

The datasets supporting this article have been uploaded as part of the electronic supplementary material.
