# Supplementary material for: Habitat and social context affect memory phenotype, exploration and covariance among these traits
Source: Philos Trans R Soc Lond B Biol Sci. 2018 Aug 13;373(1756):20170291. doi: 10.1098/rstb.2017.0291 (PMC6107572; doi:10.1098/rstb.2017.0291)
Supplement: Supplementary methods and results [file rstb20170291supp1.docx]

**Supplementary material: Dalesman.**

**Operant conditioning procedure**

Training was carried out following the procedure developed by Lukowiak et al. [1].

**Contingent training:**To test operant memory formation 500 ml of artificial pond water was placed in a 1 l glass beaker. N_2_ was then vigorously bubbled through the water for 20 min to make the water hypoxic (< 5% [O_2_]). N_2_ bubbling was reduced and continued at a low level to maintain hypoxic conditions without disturbing the animals. Snails were then introduced into the beaker in small groups of 5 to 6 individuals (except when isolated in Experiment 2) and allowed to acclimate for 10 min before the start of training. Training was carried out for 30 min (TR1), whereby the snail receives a tactile stimulus (a poke) on the pneumostome each time it attempts to open it at the water’s surface. This poke is sufficient to cause the pneumostome to close, but does not cause the snail to withdraw into its shell. The snails were then returned to aerated home aquaria for one hour and the training procedure repeated where two training sessions were required (TR2). The second training session also tests intermediate-term memory (ITM) lasting 1-3 h in *L. stagnalis*[2, 3].To test for long-term memory (LTM) the snails received an identical procedure to the training sessions 24 h following the last training session they received[2-4]. The reduction in attempted number of pneumostome openings between the first (TR1) training session and second training session (TR2) was used to determine ITM, and a reduction in attempted number of pneumostome openings between the first training session (TR1) and the memory test at 24 h (MT) session were then used to determine LTM formation among populations. Individual responses were used for analyses.

**Non-contingent (yoked) control:**To ensure that memory was formed due to operant conditioning rather than a general response to training conditions non-contingent yoked controls were carried out. Each snail in the yoked control group is randomly paired with a snail in the contingently trained group. During training, the snail in the yoked control group is then poked in the vicinity of the pneumostome (or on the pneumostome if it happens to be open at the time) when its partner is poked contingently with pneumostome opening. Therefore, the yoked animals received an identical number of stimuli during training to the contingently trained individuals. During the test phase 24 hours later, the yoked control snails receive a poke contingent with pneumostome opening. A lack of change in breathing attempts in the yoked controls allows me to determine that contingency is required for memory formation, rather than a generalized response to hypoxia or physical stimulation.

**Supplementary figure 1**

**
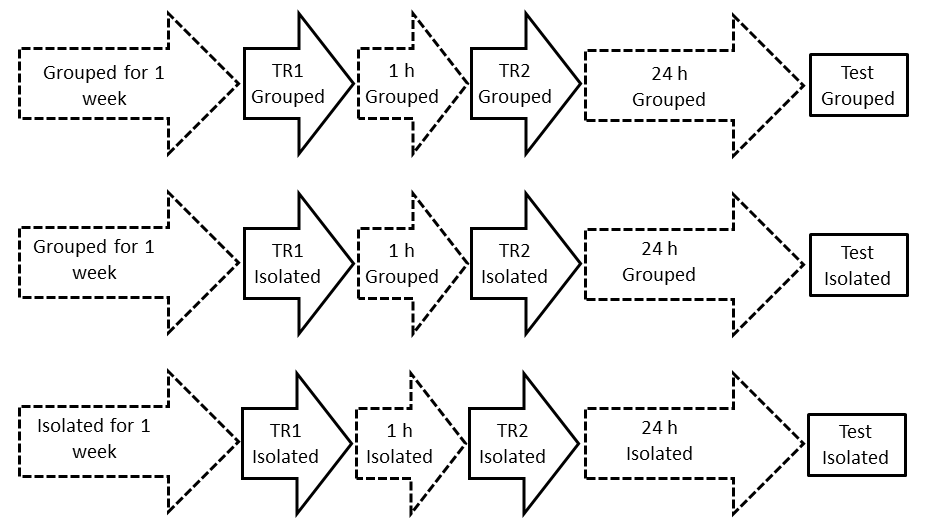
**

Figure S1: Schematic description of exposure and training protocol in assessment of the effect of isolation on memory formation (Experiment 2 in the main paper). TR1 = first training trial, TR2 = second training trial, Test = test at 24 h follow TR1. Dotted lines indicate periods when snails were maintained in aquaria.

**Supplementary figure 2**

**
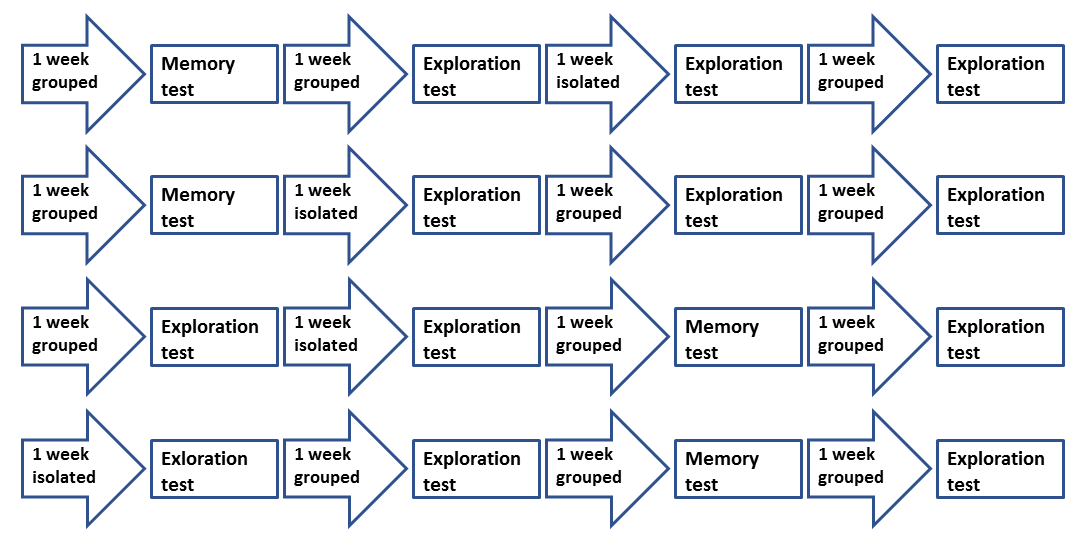
**

FigureS2: Schematic description of the order in which snails received training and exploration trials. Memory test is operant conditioning of aerial respiration.

**Supplementary Results**

Table S1: ANOVA results for long-term memory formation (proportional change in breathing rate) following: a) two half hour training sessions under grouped conditions, comparing trained vs. yoked (treatment group), habitat (laboratory vs. ditch vs. river) and population (L1, L2, D1, D2, R1, R2, R3 & R4); b) single half hour training session under grouped conditions, comparing trained vs. yoked (treatment group), habitat (laboratory vs. ditch vs. river) and population (L1, L2, D1, D2, R1, R2, R3 & R4). Significant results highlighted in bold.

| **Source** | **a) Two training sessions** | **b) Single training session** |
| --- | --- | --- |
| Habitat | F_2,5.03_ = 1.007  P = 0.429  ƞ^2^_p_ = 0.286 | F_2,5.08_ = 0.018  P = 0.982  ƞ^2^_p_ = 0.007 |
| Treatment | **F_1,5.23_ = 582.006**  **P < 0.001**  **ƞ^2^_p_ = 0.991** | **F_1,5.07_ =7.309**  **P = 0.042**  **ƞ^2^_p_ = 0.590** |
| Population(Habitat) | F_5,5_ = 3.632  P = 0.092  ƞ^2^_p_ = 0.784 | F_5,5_ = 0.931  P = 0.530  ƞ^2^_p_ = 0.482 |
| Habitat*treatment | F_2,5.09_ =3.011  P = 0.137  ƞ^2^_p_ = 0.542 | F_2,5.07_ = 0.087  P = 0.918  ƞ^2^_p_ = 0.033 |
| Population(Habitat)*treatment | F_5,172_ = 0.147  P = 0.981  ƞ^2^_p_ = 0.004 | **F_5,190_ = 2.789**  **P = 0.019**  **ƞ^2^_p_ = 0.068** |

Table S2: Post-hoc comparison of proportional change in breathing response between contingently trained and yoked (non-contingent) snails in each population following a single half hour training session under grouped conditions. Significant comparisons indicated in bold (Sidak P < 0.05).

| Population | Mean difference  (yoked – contingent) | P-value | 95% Confidence interval of the difference |
| --- | --- | --- | --- |
| Laboratory 1 | **-0.702** | **P = 0.002** | **-1.142, -0.261** |
| Laboratory 2 | -0.149 | P = 0.454 | -0.539, 0.242 |
| Ditch 1 | **-0.619** | **P = 0.001** | **-0.985, -0.254** |
| Ditch 2 | 0.028 | P = 0.905 | -0.434, 0.490 |
| River 1 | -0.153 | P = 0.476 | -0.574, 0.269 |
| River 2 | -0.018 | P = 0.928 | -0.408, 0.372 |
| River 3 | **-0.667** | **P = 0.002** | **-1.088, -0.245** |
| River 4 | **-0.418** | **P = 0.036** | **-0.808, -0.028** |

Table S3: ANOVA results for the effect of isolation treatment (treatment: grouped vs. isolated during training and testing only vs. isolated for 1 week), habitat (laboratory vs. ditch vs. river) and population (L1, L2, D1, D2, R1, R2, R3 & R4) on: a) initial breathing rate during the first training session; b) proportional change in breathing behaviour (intermediate-term memory during the second training trial 1 h following the first trial; and c) proportional change in breathing behaviour (long-term memory) during the test at 24 h. Significant results highlighted in bold.

| **Source** | **a) initial breathing rate** | **b) intermediate-term memory** | **c) long-term memory** |
| --- | --- | --- | --- |
| Habitat | F_2,4.95_ = 0.206  P = 0.820  ƞ^2^_p_ = 0.077 | **F_2,4.68_ = 9.644**  **P = 0.022**  **ƞ^2^_p_ = 0.805** | F_2,4.99_ = 0.153  P = 0.862  ƞ^2^_p_ = 0.058 |
| Treatment | F_2,9.96_ = 0.230  P = 0.799  ƞ^2^_p_ = 0.044 | F_2,9.94_ =2.453  P = 0.136  ƞ^2^_p_ = 0.331 | **F_2,9.99_ = 5.377**  **P = 0.026**  **ƞ^2^_p_ = 0.518** |
| Population(Habitat) | F_5,10.28_ = 1.024  P = 0.452  ƞ^2^_p_ = 0.333 | F_5,10.45_ = 0.250  P = 0.931  ƞ^2^_p_ = 0.107 | F_5,10.04_ = 1.021  P = 0.455  ƞ^2^_p_ = 0.337 |
| Habitat*treatment | F_4,9.91_ = 1.704  P = 0.226  ƞ^2^_p_ = 0.408 | F_4,9.85_ = 2.181  P = 0.146  ƞ^2^_p_ = 0.470 | F_4,9.99_ = 0.195  P = 0.935  ƞ^2^_p_ = 0.073 |
| Population(Habitat)*  treatment | F_10,306_= 0.504  P = 0.887  ƞ^2^_p_ = 0.016 | F_10,306_= 0.314  P = 0.977  ƞ^2^_p_ = 0.010 | **F_10,306_= 3.256**  **P = 0.001**  **ƞ^2^_p_ = 0.096** |

Intermediate-term memory:

Habitat had a significant overall effect on intermediate-term memory formation, with snails from ditch (-0.485 ± 0.041) and river (-0.500 ± 0.029) populations showing a trend towards a greater proportional reduction in mean (± SEM) breathing rate compared to laboratory populations (-0.441 ± 0.040). However, post-hoc pair-wise comparisons among habitats were not significant (Sidak: laboratory vs. ditch: P = 0.826; laboratory vs. river: P = 0.535; ditch vs. river: P = 0.986).

Table S4: Pair-wise comparisons of proportional change in breathing attempts when tested at 24 h for long-term memory between: grouped snails and snails isolated during training only (Training only); and grouped snails and snails isolated for one week and during training (1-week isolation) from each population. Significant comparisons indicated in bold (Sidak P < 0.05).

| Population | Isolation condition compared to grouped response | Mean difference  (isolated – grouped) | P-value | 95% Confidence interval of the difference |
| --- | --- | --- | --- | --- |
| Laboratory 1 | Training only | -0.02 | 0.999 | -0.445, 0.399 |
|  | **1-week isolation** | **-0.686** | **< 0.001** | **-1.108, -0.264** |
| Laboratory 2 | Training only | 0.087 | 0.946 | -0.338, 0.513 |
|  | 1-week isolation | -0.040 | 0.995 | -0.471, 0.391 |
| Ditch 1 | Training only | -0.097 | 0.943 | -0.562, 0.367 |
|  | **1-week isolation** | **-0.808** | **<0.001** | **-1.244, -0.372** |
| Ditch 2 | Training only | -0.069 | 0.975 | -0.511, 0.373 |
|  | 1-week isolation | -0.031 | 0.998 | -0.473, 0.411 |
| River 1 | Training only | -0.179 | 0.687 | -0.610, 0.253 |
|  | 1-week isolation | -0.044 | 0.993 | -0.475, 0.388 |
| River 2 | Training only | 0.008 | > 0.999 | -0.428, 0.445 |
|  | 1-week isolation | -0.008 | > 0.999 | -0.450, 0.434 |
| River 3 | Training only | -0.051 | 0.989 | -0.483, 0.380 |
|  | **1-week isolation** | **-0.551** | **0.007** | **-0.983, -0.120** |
| River 4 | Training only | -0.095 | 0.944 | -0.550, 0.360 |
|  | **1-week isolation** | **-0.487** | **0.024** | **-0.925, -0.049** |

**Supplementary Figure 3**


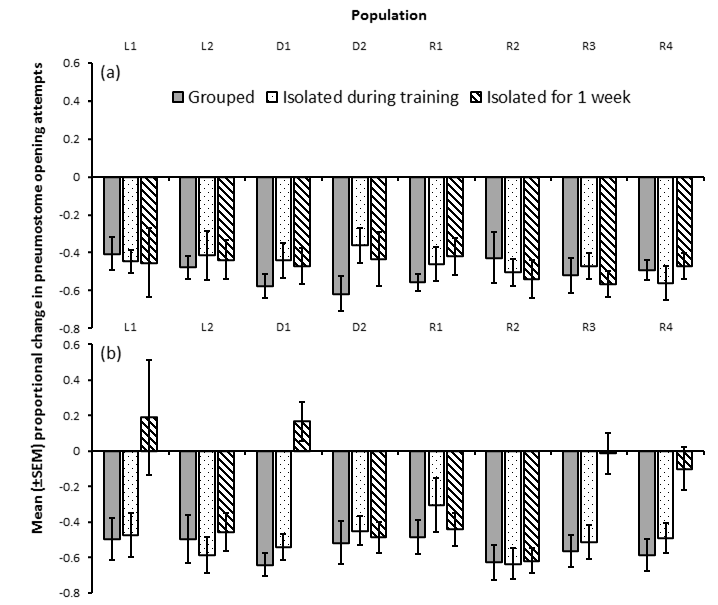


Figure S3: Mean proportional change in pneumostome opening attempts compared to the first training trial across eight populations either grouped throughout, isolated during training only or isolated for a week: a) intermediate-term memory (during the second training trial) and b) long-term memory (during the test at 24 h following two-trial training).

**Plasticity in exploration traits**

**Data analyses**

Exploration traits, speed and thigmotaxis, were analysed using repeated measures ANOVA (rmANOVA). Memory phenotype was allocated based on results on single trial training from Experiment 1; populations that had failed to demonstrate LTM formation were designated 'Phenotype A' (populations L2, D2, R1 and R2) and those that did demonstrate LTM were designated 'Phenotype B' (populations L1, L2, R3 and R4). Social condition (grouped vs. isolated) was used as the within-subject factor, between-subject factors were habitat of origin (laboratory vs. ditch vs. river) and memory phenotype (phenotype A vs. phenotype B) were used as fixed factors and population as a random factor, nested in habitat*memory phenotype.

**Results**

The effect of isolation on crawling speed was dependent on memory phenotype (Fig. S4a; rmANOVA: memory phenotype*isolation: F_1,144_ = 10.669, P = 0.001, ƞ^2^_p_ = 0.069; Table S5). Phenotype A snails (no LTM following single trial training) did not significantly alter their crawling speed in response to isolation (Sidak: P = 0.306, mean difference = -0.036 mm/s, CI -0.105, 0.033); whereas phenotype B snails (LTM formed following single trial training) significantly reduced crawling speed following isolation (Sidak: P < 0.001, mean difference = -0.209 mm/s, CI -0.278, -0.140). The crawling speed of different memory phenotypes was also dependent on habitat of origin (Fig. S4a; rmANOVA: memory phenotype*habitat: F_2,144_ = 7.198, P = 0.001, ƞ^2^_p_ = 0.091; Table S5). There was no overall difference in crawling speed between phenotypes A and B originating from ditch habitats (Sidak: P = 0.967, mean difference = 0.003 mm/s, CI -0.124, 0.129); however, phenotype B snails originating from laboratory strains crawled faster overall compared to phenotype A (Laboratory: Sidak: P = 0.024, mean difference = 0.150 mm/s, CI 0.020, 0.280), conversely, phenotype B snails originating from river strains crawled slower overall compared to phenotype A (River: Sidak: P = 0.001, mean difference = -0.146 mm/s, CI -0.234, -0.058).

The effect of isolation on thigmotaxis depended on both memory phenotype and habitat (Fig. S4b; rmANOVA: habitat*memory phenotype*isolation: F_2,144_ = 3.294, P = 0.040, ƞ^2^_p_ = 0.044; Table S5). In laboratory populations the proportion of time in thigmotaxis reduced following isolation for both memory phenotypes (Phenotype A: Sidak: P = 0.029, mean difference -0.127, CI -0.241, -0.014; Phenotype B: Sidak: P < 0.001, mean difference -0.220, CI -0.334, -0.106). Ditch populations only showed a significant reduction in thigmotaxis following isolation in phenotype B snails (Phenotype A: Sidak: P = 0.873, mean difference 0.009, CI -0.102, 0.120; Phenotype B: Sidak: P < 0.001, mean difference -0.363, CI -0.473, -0.252). Likewise, river populations also only showed a significant reduction in thigmotaxis following isolation in phenotype B snails (Phenotype A: Sidak: P = 0.589, mean difference -0.021, CI -0.098, 0.056; Phenotype B: Sidak: P < 0.001, mean difference -0.316, CI -0.393, -0.239).

Table S5: Repeated measures ANOVA results for crawling behaviour, showing results for crawling speed (a) and thigmotaxis (b). Within-subject: social condition (grouped vs. isolation); between-subject factors: habitat (laboratory vs. ditch vs. river); memory phenotype (A vs. B) and population (L1, L2, D1, D2, R1, R2, R3 & R4, nested in habitat*memory phenotype). Significant results highlighted in bold.

| **Source** | **a) Crawling speed** | **b) Thigmotaxis** |
| --- | --- | --- |
| **Within-subject comparisons:** | | |
| social condition | **F_1,144_ = 21.776**  **P < 0.001**  **ƞ^2^_p_ = 0.131** | **F_1,144_ = 69.956**  **P < 0.001**  **ƞ^2^_p_ = 0.327** |
| social condition  *habitat | F_2,144_ = 2.013  P = 0.137  ƞ^2^_p_ = 0.027 | F_2,144_ = 0.016  P = 0.984  ƞ^2^_p_< 0.001 |
| social condition  *memory phenotype | **F_1,144_ = 10.699**  **P = 0.001**  **ƞ^2^_p_ = 0.069** | **F_1,144_ = 40.162**  **P < 0.001**  **ƞ^2^_p_ = 0.218** |
| social condition  *population (memory phenotype*habitat) | F_2,144_ = 1.299  P = 0.296  ƞ^2^_p_ = 0.017 | F_2,144_ = 1.659  P = 0.194  ƞ^2^_p_ = 0.023 |
| social condition  *memory phenotype  *habitat | F_2,144_ = 0.949  P = 0.389  ƞ^2^_p_ = 0.013 | **F_2,144_ = 3.294**  **P = 0.040**  **ƞ^2^_p_ = 0.044** |
| **Between-subject comparisons** | | |
| habitat | F_2,144_ = 0.955  P = 0.387  ƞ^2^_p_ = 0.013 | F_2,144_ = 0.315  P = 0.730  ƞ^2^_p_ = 0.004 |
| memory phenotype | F_1,144_ = 0.146  P = 0.703  ƞ^2^_p_ = 0.001 | **F_1,144_ = 14.144**  **P < 0.001**  **ƞ^2^_p_ = 0.089** |
| population  (habitat*memory phenotype) | F_2,144_ = 0.987  P = 0.375  ƞ^2^_p_ = 0.014 | F_2,144_ = 2.464  P = 0.089  ƞ^2^_p_ = 0.033 |
| habitat*memory phenotype | **F_2,144_ = 7.198**  **P = 0.001**  **ƞ^2^_p_ = 0.091** | **F_2,144_ = 5.603**  **P = 0.005**  **ƞ^2^_p_ = 0.072** |

**Supplementary figure 4**

**
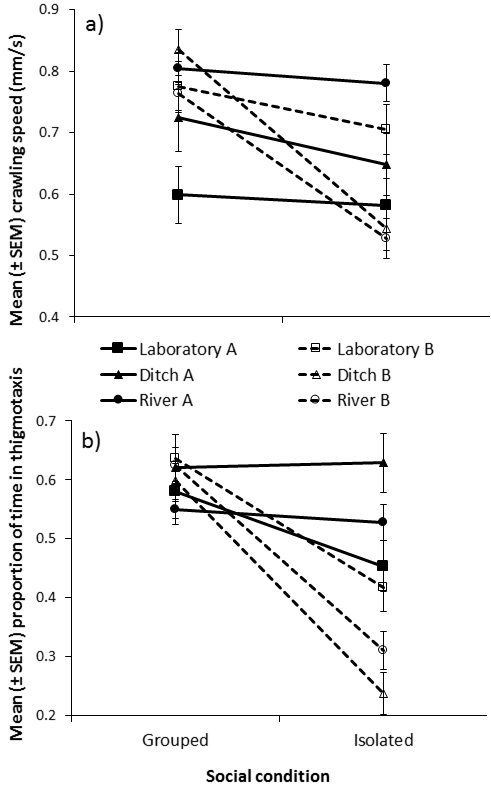
**

Figure S4: Exploration behaviour in grouped and isolated conditions: a) crawling speed; and b) proportion of time spent in thigmotaxis. Phenotype A = no LTM following single trial training (Laboratory: L2; Ditch: D2; River: R1 and R2); phenotype B = LTM is formed following single trial training (Laboratory:L1; Ditch: D1: River: R3 and R4).

Supplementary material references:

[1] Lukowiak, K., Ringseis, E., Spencer, G., Wildering, W. & Syed, N. 1996 Operant conditioning of aerial respiratory behaviour in *Lymnaea stagnalis*. *Journal of Experimental Biology***199**, 683-691.

[2] Sangha, S., Scheibenstock, A., McComb, C. & Lukowiak, K. 2003 Intermediate and long-term memories of associative learning are differentially affected by transcription versus translation blockers in *Lymnaea*. *The Journal of Experimental Biology***206**, 1605-1613. (doi:DOI: 10.1242/jeb.00301 ).

[3] Lukowiak, K., Adatia, N., Krygier, D. & Syed, N. 2000 Operant conditioning in *Lymnaea*: Evidence for intermediate- and long-term memory. *Learning & Memory***7**, 140-150. (doi:10.1101/lm.7.3.140 ).

[4] Scheibenstock, A., Krygier, D., Haque, Z., Syed, N. & Lukowiak, K. 2002 The soma of RPeD1 must be present for long-term memory formation of associative learning in *Lymnaea*. *Journal of Neurophysiology***88**, 1584-1591. (doi:10.1152/jn.00258.2002).
